# Effects of Antioxidant Supplements on the Survival and Differentiation of Stem Cells

**DOI:** 10.1155/2017/5032102

**Published:** 2017-07-09

**Authors:** Sara Shaban, Mostafa Wanees Ahmed El-Husseny, Abdelrahman Ibrahim Abushouk, Amr Muhammad Abdo Salem, Mediana Mamdouh, Mohamed M. Abdel-Daim

**Affiliations:** ^1^Faculty of Medicine, Fayoum University, Cairo, Egypt; ^2^NovaMed Medical Research Association, Cairo, Egypt; ^3^Faculty of Medicine, Ain Shams University, Cairo, Egypt; ^4^Medical Research Group of Egypt, Cairo, Egypt; ^5^Pharmacology Department, Faculty of Veterinary Medicine, Suez Canal University, Ismailia 41522, Egypt; ^6^Department of Ophthalmology and Micro-Technology, Yokohama City University, Yokohama, Japan

## Abstract

Although physiological levels of reactive oxygen species (ROS) are required to maintain the self-renewal capacity of stem cells, elevated ROS levels can induce chromosomal aberrations, mitochondrial DNA damage, and defective stem cell differentiation. Over the past decade, several studies have shown that antioxidants can not only mitigate oxidative stress and improve stem cell survival but also affect the potency and differentiation of these cells. Further beneficial effects of antioxidants include increasing genomic stability, improving the adhesion of stem cells to culture media, and enabling researchers to manipulate stem cell proliferation by using different doses of antioxidants. These findings can have several clinical implications, such as improving neurogenesis in patients with stroke and neurodegenerative diseases, as well as improving the regeneration of infarcted myocardial tissue and the banking of spermatogonial stem cells. This article reviews the cellular and molecular effects of antioxidant supplementation to cultured or transplanted stem cells and draws up recommendations for further research in this area.

## 1. Introduction

Stem cells are undifferentiated cells, characterized by self-renewal and the ability to differentiate into several cell types (potency) [1]. They can be totipotent (differentiating into embryonic and extraembryonic cell types), pluripotent (differentiating into cells of the three germ layers), or multipotent (differentiating into cells of a closely related family) [2]. Stem cell research runs with an incredible speed and its applications are under investigation in different medical fields [3, 4]. There are two main types of stem cells: embryonic stem cells (ESCs) (present in the inner cell mass of the blastocyst) and adult stem cells (present in different mature tissues to replace dead cells) [5, 6].

Induced pluripotent stem cells (iPSCs) are adult cells, genetically reprogrammed to express genes and factors, required for maintaining the properties of ESCs. However, the reprogramming process itself results in oxidative stress by generating high levels of reactive oxygen species (ROS) [7, 8], which cause damage to DNA, RNA, and cell proteins and may induce apoptosis [9–11]. However, ROS are required in physiological levels to maintain the self-renewal capacity of stem cells and to fight invading microbes [11–14].

Antioxidants are biochemical supplements that protect cellular constituents from oxidative stress by neutralizing free radicals and terminating the oxidative reaction chain in the mitochondrial membrane [15]. They can be classified into enzymatic and nonenzymatic, endogenous and exogenous [16], and water-soluble (reacting with oxidants in the cytosol or plasma) and lipid-soluble antioxidants (preventing lipid peroxidation of cell membranes) [17].

Over the past decade, several studies have shown that antioxidants can not only mitigate oxidative stress and improve stem cell survival but also affect the potency and differentiation of these cells. In our article, we reviewed the results of preclinical studies that investigated the effects of antioxidants on cultured or transplanted stem cells in an attempt to draw up recommendations for further research in this area.

## 2. Induced Pluripotent Stem Cells (iPSCs)

As highlighted earlier, the reprogramming of iPSCs is associated with generation of high ROS levels. Several reports showed that, in comparison to somatic precursor cells, iPSCs exhibit the following criteria: (1) marked protection against nuclear and mitochondrial DNA (mtDNA) damage and (2) significantly lower levels of ROS due to upregulation of intrinsic antioxidant enzymes [18, 19]. Dannenmann et al. found a 10-fold decrease in ROS level and a fourfold increase of glutathione (GSH) and glutathione reductase (GR) levels in iPSCs, compared to fibroblasts [18]. In another study by the same authors, they showed that several glutathione S-transferases (GSTs), which act as antioxidant and detoxifying enzymes, were upregulated in iPSCs, compared to their somatic precursor cells [19].

Ji and colleagues reported that mitigation of oxidative stress during cellular reprogramming by antioxidant supplementation protects the genome of reprogramming cells against DNA damage and leads to iPSCs with fewer genomic aberrations [20]. In the same vein, Luo and colleagues [21] found that iPSCs grew well and “stemness” was preserved for up to two months after the addition of a low-dose antioxidant supplement. Moreover, using comparative genomic hybridization (CGH) analysis, they showed that antioxidant supplementation lowered the levels of genetic aberrations in cultured iPSCs [21].

Hämäläinen and colleagues showed that the reprogramming and self-renewal abilities of iPSCs were diminished after subtle increases in ROS levels, originating from mtDNA mutagenesis. However, the addition of two different antioxidants [N-acetyl-L-cysteine (NAC) and mitochondria-targeted ubiquinone (MitoQ)] efficiently rescued these abilities in mutator iPSCs [22]. N-acetyl-L-cysteine raises cellular GSH pool and promotes the processing of H_2_O_2_ in the cytosol [23], whereas MitoQ acts upstream to prevent superoxide production within the mitochondria before H_2_O_2_ generation [24]. Of note, Hämäläinen et al. highlighted that the therapeutic window of MitoQ for iPSCs is narrow, while high concentrations of NAC were not associated with toxic effects on iPSCs [22].

Interestingly, other reports showed no effect of antioxidant supplementation on the expression of 53BP1 and ATM proteins (two molecules involved in DNA repair pathways) [25–27]. Recently, it has been found that high-dose antioxidants downregulates DNA repair-related kinases, which conversely results in genomic instability of iPSCs [21]. Therefore, adjusting the dose of supplementary antioxidants is critical.

## 3. Bone Marrow-Derived Mesenchymal (BMSCs) and Hematopoietic Stem Cells (HSCs)

Several studies showed that the ex vivo expansion of ESCs and mesenchymal stem cells (MSCs) [28–31] and the in vitro expansion of HSCs [32] may cause genomic instability. Through a serial transplantation assay, Jang and colleagues showed that elevated ROS levels reduce the self-renewal ability of HSCs [33]. Therefore, decreasing O_2_ concentrations to physiological levels or adding proper dosages of antioxidants can reduce in vitro, culture-stimulated aneuploidy, providing potential methods to limit genomic alterations when expanding HSCs in vitro [32, 34, 35]. Hamid et al. conducted an in vitro study to evaluate the antioxidant effects of *Hibiscus sabdariffa* L. (roselle) on bone marrow-derived HSCs. They showed that roselle supplementation increased superoxide dismutase (SOD) expression (at 125, 500, and 1000 ng/mL) and HSCs survival (at 500 and 1000 ng/mL) and protected against H_2_O_2_-induced DNA damage [36].

In another study by Halabian et al., treatment of BMSCs with Lipocalin-2 (Lcn2), a natural cytoprotective factor generated upon exposure to stressful conditions, increased cellular resistance against oxidative, hypoxic, and serum deprivation stresses. Moreover, Lcn2-treated cells showed SOD gene upregulation, increased proliferation, maintained pluripotency, and improved cellular adhesion to culture media upon H_2_O_2_ exposure, in comparison to untreated cells [37]. Similarly, Fan and colleagues studied different methods for isolation of BMSCs, aiming at reducing the number of chromosomal abnormalities in isolated cells. They reported that culturing isolated BMSCs at a low O_2_ concentration (2%) or with antioxidant (NAC) supplementation increased cellular proliferation and genomic stability, in comparison to cultured cells at normoxic concentrations (20% O_2_) [38].

Another study by Choi et al. demonstrated that adding ascorbic acid 2-phosphate (AAP) at different concentrations can influence the fate of BMSCs, that is, AAP significantly increased osteogenic differentiation at 50 mM concentration, while a significant induction of adipogenic differentiation with oil droplet formation was noted at concentrations of 250 mM and higher [39].

## 4. Cardiomyoblasts and Vascular Progenitor Cells

According to Li and colleagues, culturing cardiac stem cells with antioxidant increased the number and severity of cytogenic abnormalities. This could be explained by the excessive decrease in ROS to subphysiological levels, which may downregulate DNA repair enzymes [40]. In another study by Rodriguez-Porcel et al., modulation of the microenvironment, using antioxidants, leads to a higher rate of cardiomyoblast survival, early after transplantation to the myocardium of small animals [41]. Therefore, oxidative stress blockade may provide a favorable microenvironment for stem cells' engraftment and survival in the heart [42].

Song and colleagues reported increased ROS production during differentiation of human ESCs into vascular progenitor cells (CD34+ cells) due to increased activity of NADPH oxidase-4 (Nox4) enzyme. They found that moderate ROS scavenging, using selenium, enhanced the vascular differentiation of human ESCs, while complete ROS scavenging, using NAC, totally inhibited the vascular differentiation of these cells. This confirms that a minimal level of ROS is required for vascular stem cell differentiation to occur [43].

## 5. Neural Stem Cells (NSCs)

Neural stem cells are multipotent stem cells that have been suggested as a therapeutic agent to enhance the recovery of injured tissues in neuroinflammatory diseases [44]. Park and colleagues tested the effects of GV1001, a novel antioxidant agent, derived from human telomerase reverse transcriptase, on in vitro-cultured mouse NSCs. They showed that GV1001 treatment attenuated the effects of H_2_O_2_ exposure, reduced lipid peroxidation and mtDNA mutation, and induced the expression of survival-related proteins [45]. Hachem et al. reported that treatment of NSCs, isolated from the spinal cords of transgenic mice, with brain-derived neurotrophic factor improved cell viability by increasing the levels of GR and SOD enzymes; however, it had no effect on cellular proliferation [46].

Nitric oxide (NO) and nitric oxide synthase (NOS)-dependent signaling pathways have been implicated in different neurodegenerative diseases [47, 48]. Moreover, NO levels were linked to neural precursor cell (NPC) survival and cell fate determination [49], that is, elevated levels of NO suppress NSC proliferation and enhance differentiation of NPCs into astrocytes [50, 51]. Melatonin is a hormone synthesized in the pineal gland [52] with indirect antioxidant abilities through induction of antioxidant enzymes [53] and inhibiting NO production in glial cultures through p38 inhibition [54]. It has been shown to protect NSCs against lipopolysaccharide- (LPS-) induced inflammation [52]. Moreover, Negi et al. demonstrated that melatonin mitigates neuroinflammation and oxidative stress via upregulating nuclear factor (erythroid-derived 2) (Nrf2) [55], a transcription factor which stimulates the PI3K-Akt survival signaling pathway [56, 57] and increases the expression of the antioxidant enzyme heme oxygenase-1 (HO-1) [55].

To test the effects of in vitro antioxidant supplementation, Petro et al. divided male rats with experimentally induced thromboembolic stroke into four groups: normal rats, untreated rats with stroke, rats receiving tissue plasminogen activator (tPA) only, and rats receiving tPA + CAT/SOD (loaded on nanoparticles) at three hours post stroke. Two days later, brain tissue samples were harvested for analysis. Brain sections from the untreated group showed evidence of NSC migration through the rostral migratory stream (through detection of NSCs markers, such as nestin, GFAP, and SOX2), confirming the occurrence of neurogenesis following stroke. However, brain tissue samples from the tPA-alone group showed reduction in NSCs migration, indicating that tPA treatment suppresses neurogenesis, either directly or through reperfusion-induced ROS generation injury. Interestingly, tPA + Nano-CAT/SOD treatment restored and significantly increased NSCs migration [58].

## 6. Human Adipose-Derived Stem Cells (ADSCs)

Adipose-derived stem cells are multipotent stem cells that can be isolated from the human adipose tissue and are capable of in vitro expansion. Sun and colleagues reported that both hypoxia and antioxidants promoted ADSCs proliferation by raising the number of cells in the S phase, but the maximal increase in cell number was produced in the presence of antioxidants [59]. Hypoxia is believed to influence the secretion of several growth factors [60, 61], such as insulin-like growth factor and hepatocyte growth factor [62], while antioxidants increase the expression of stemness genes (CDK2, CDK4, and CDC2) and the differentiation potential of ADSCs [59]. Another study by Higuchi et al. found that lentivirus-mediated NADPH oxidase-4 (Nox-4) overexpression did not increase ROS production in insulin, dexamethasone, indomethacin, and 3-isobutyl-1-methylxanthine (IDII)-stimulated ADSCs [63]. This finding was later explained by the increased expression of endogenous antioxidants, such as SOD and CAT during adipogenesis [63, 64].

Yang et al. showed that treatment of ADSCs with fullerol (a polyhydroxylated fullerene) potentiated the expression of the transcription factor FoxO1 and its downstream genes, such as Runx2 and SOD2. Moreover, it enhanced the osteogenic activity of ADSCs, as evidenced by increased mineralization and expression of osteogenic markers (Runx2, OCN, and alkaline phosphatase) [65]. Wang and colleagues showed that pretreatment of ADSCs with NAC (3 mM) or AAP (0.2 mM) for 20 hours suppressed advanced glycosylation end product- (AGE-) induced apoptosis via a microRNA-dependent mechanism by inhibiting AGE-induced overexpression of miRNA-223: a key modulator of intracellular apoptotic signaling [66].

## 7. Human Periodontal Ligament Cells (hPDLCs)

In a recent study, Chung and colleagues showed that treating hPDLCs with deferoxamine (DFO), an iron chelator, results in a dose-dependent elevation in ROS levels, 24 hours after treatment [67]. The same finding was reported in rabbit cardiomyocytes [68] and normal human hepatocytes [69]. However, DFO has the ability to act on Nrf2, increasing its nuclear translocation and the expression of its target genes, including GST and glutamate cysteine ligase (GCL) [67]. Therefore, DFO has both beneficial (Nrf2-mediated antioxidant effect) and cytotoxic (increased ROS levels) effects. GSH depletion, using buthionine sulfoximine (BSO) and diethyl maleate (DEM), was shown to inhibit DFO-stimulated hPDLC differentiation into osteoblasts [67]. Moreover, GSH depletion was also reported to repress myogenic differentiation of murine skeletal muscle (C2C12) cells [70] and phorbol-12-myristate-13-acetate- (PMA-) stimulated differentiation of human myeloid cell line (HL-60) [71].

## 8. Muscle-Derived Stem Cells (MDSCs)

According to Drowley and colleagues, injection of injured skeletal muscles with NAC-treated MDSCs significantly increased muscle regeneration, compared to muscles injected with untreated or DEM-treated MDSCs. The direction of scar tissue formation was opposite the direction of the host muscle regeneration [72]. Additionally, they showed an improved survival of NAC-treated MDSCs, probably due to stimulation of extracellular signal-regulated kinase (ERK) pathway, as evidenced by decreased survival of NAC treated cells after inhibition of the ERK pathway [72, 73].

Moreover, they demonstrated that experimentally infarcted hearts, injected with NAC-treated MDSCs, showed a more significant reduction in the percentage area of collagenous scar tissue than hearts injected with either untreated, DEM-treated, or phosphate buffered saline- (PBS-) treated MDSCs. There was no difference in myocardial scar formation between hearts injected with DEM-treated MDSCs and those injected with PBS [72].

## 9. Spermatogonia Stem Cells (SSCs)

Cryopreservation of spermatogonial stem cells, in the presence of catalase (CAT) and *α*-tocopherol (*α*-TCP), promoted cell viability and suppressed apoptosis through inducing the expression of the antiapoptotic BcL-2 gene and inhibiting the expression of the proapoptotic BAX gene [74]. In other studies, cryopreservation with antioxidants could promote cell enrichment and increase the efficiency of colony formation in isolated SSCs [75, 76]. Spermatogonia-derived colonies showed increased SSC marker activity, enhanced expression of self-renewal genes, such as promyelocytic leukemia zinc finger (Plzf) protein and DNA-binding protein inhibitor ID4, and suppressed expression of the proto-oncogene (c-kit) in both CAT and *α*-TCP treated groups [74]. This technique can increase the possibility of SSCs banking for men with malignant diseases and promote the resumption of spermatogenesis in SCCs recipients. A summary of the design and main findings of included studies is illustrated in Table 1.

## 10. Discussion

**Figure 1:**
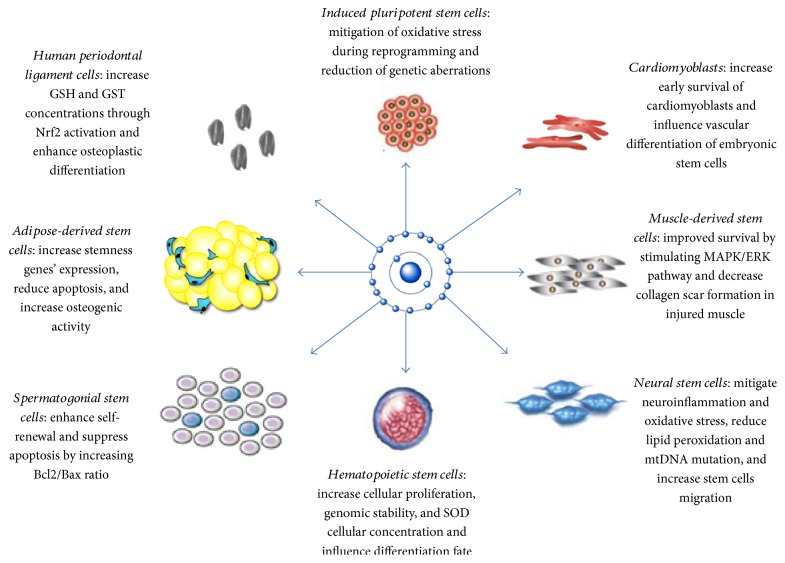
Summarizes the effects of antioxidants on different types of stem cells.

Our review highlights that antioxidants can influence stem cell activities by [1] mitigating oxidative stress through neutralization of free radicals and increasing the expression of antioxidant enzymes and [2] influencing the differentiation fate of precursor stem cells. Further beneficial effects of antioxidant treatment include increasing genomic stability, improving the adhesion of stem cells to culture media, and enabling researchers to manipulate stem cell proliferation by using different doses of antioxidants.  Figure 1 summarizes the effects of antioxidants on different types of stem cells.

We also discussed that a physiological level of ROS (oxidative optimum) is needed for proper differentiation of stem cells, especially for proper cardiogenesis and vasculogenesis [40]. These findings can have several clinical applications, such as improving neurogenesis in patients with stroke and neurodegenerative diseases, as well as improving the regeneration of infarcted myocardial tissue and the banking of SCCs.

Antioxidants are prevalent supplements worldwide. However, little is known about their cell-type-specific actions. It has been shown that a therapeutic dose may vary between different cell types: a dose that rescues a pathology in one tissue may roughly challenge the function of another [22]. Therefore, there is a need for dose-effect studies on antioxidants to confirm their safety as nutritional supplements or therapeutic agents—particularly in the case of antioxidants accumulating in the mitochondria. Our review also showed the potential of some endogenous molecules, such as melatonin, BDNF, and the adipokine (lipocalin-2) in preserving stem cell viability and differentiation potential. Whether these compounds can be used in future clinical applications of stem cells and whether other endogenous molecules with proven antioxidant activities, such as adiponectin [84], can be useful in this regard require further investigation.

## 11. Recommendations

1. Multiplicity of stem cell sources within the body (different home environments) and their variable ROS scavenging capacity make them susceptible to oxidative stress at different thresholds. Therefore, we tried to review each stem cell type as a separate entity and we believe that clearing those differences on the molecular and genetic levels will optimize the clinical application of stem cells in different medical fields.
2. Most of stem cell characteristics are established within in vitro culturing environments. More in vivo studies are required to define their interactions within the body. Furthermore, few in vivo studies have focused on the long-term survival of transplanted stem cells; therefore, this should be the interest of future studies.
3. The effect of ROS level and redox state on the long-term oncogenicity of stem cells should be further investigated prior to in vivo clinical trials.

## 12. Conclusion

Using antioxidants can improve the viability and self-renewal capacity of stem cells and affect their differentiation potential. More research is needed on the dose-effect association and cell-type-specific actions of antioxidant before applying these findings in human therapeutic trials.

## Figures and Tables

**Table 1:** Summary of the results of in vitro and in vivo studies on treatment of stem cells with antioxidant supplements.

Study ID	Antioxidant (dose)	Stem cell type (source)	Findings	Possible mechanisms
Ji et al. [20]	N-Acetyl-L-cysteine (NAC) and vitamin C.	Induced pluripotent stem cells (iPSCs) generated from human neonatal foreskin fibroblasts.	In cells, infected with reprogramming factors (retroviruses encoding human OCT4, SOX2, KLF4, and c-MYC), supplementation of the culture media with NAC significantly increased iPSCs survival and reduced ROS generation and the number of DNA double-stranded breaks in the reprogrammed cells.	Antioxidants significantly reduced ROS generation and the number of copy-number variations (CNVs: an indication of genomic aberrations) in treated iPSCs, compared to the untreated control group (*p* < 0.02). Treatment with NAC had no effect on transgene expression, silencing, and viral transduction efficiency.
Luo et al. [21]	Homemade antioxidant cocktail [ascorbate, glutathione, and *α*-tocopherol at 20 mM, 4 mM, and 1 mM, resp.].	Two human cell lines of iPSCs (201B7 and 253G1).	(i) Measurement of cellular ROS levels showed diminished ROS levels in cells, cultured with antioxidants, compared to the untreated group. (ii) Moreover, the addition of homemade antioxidant cocktail reduced the number of genomic aberrations in treated cells.	(i) The components of homemade cocktail exerted a free-radical scavenging activity to neutralize ROS in treated cells. (ii) For two months of culturing with low doses of antioxidants, iPSCs maintained the expression of stemness-related genes (Oct3/4, Nanog, SSEA-4, and ALP).
Hamid et al. [36]	Roselle (*Hibiscus sabdariffa* L.) at 125, 500, or 1000 ng/mL.	Bone marrow-derived hematopoietic stem cells (HSCs) from murine bone marrow.	(i) Adding roselle (at 500 and 1000 ng/mL) significantly increased the survival of HSCs and protected them against H_2_O_2_-induced DNA damage. (ii) Roselle supplementation was genoprotective, as evidenced by the nonremarkable difference on the percentage of tail DNA, compared to the control group (untreated BMSCs).	Compared to the control group, roselle enhanced the activity of SOD in HSCs (at 125, 500, and 1000 ng/mL) with a significant increase in GSH level (*p* < 0.05). However, there was no difference in ROS levels between roselle-treated and control groups.
Ikeda et al. [77]	Poly(ethylene glycol)-b-poly[4-(2,2,6,6-tetramethylpiperidine-1-oxyl)amino-methylstyrene] (PEG-b-PMNT).	Hematopoietic stem cells (HSCs) from mice fetal liver cells.	Ikeda et al. designed a biocompatible cell culture surface that can be used during ex vivo culturing and expansion of HSCs. This new surface has several advantages, compared to the currently used one including low molecular weight and antioxidant supplementation. It decreased ROS production, inhibited apoptosis, and increased the purity of separated cells.	The antioxidant culture surface (PEG-b-PMNT) scavenged nitric oxide radicals and reduced oxidative membrane damage without changing the mitochondrial membrane potential because it is not internalized within the cell as the conventional LMW systems.
Liu et al. [32]	N-Acetyl-L-cysteine (NAC) at 0.1 to 1 *μ*M.	LSK cells (Lin− Sca-1+ c-Kit+, a population enriched with HSCs).	(i) Aneuploidy/chromosomal instability during in vitro expansion of HSCs is ROS-mediated and can be minimized by maintaining a hypoxic condition (3% O_2_) during cell culturing. (ii) Similarly, NAC administration significantly reduced the percentage of aneuploidy in HSCs, cultured under normoxic conditions, only at low dosages (0.1 *μ*M).	(i) At optimum concentrations, NAC significantly reduced oxidative damage due to its ROS-scavenging activity. (ii) Moreover, hypoxic conditions and NAC reduced the percentage of aneuploidy in both young and old aged stem cells.
Halabian et al. [37]	Lipocalin-2 (Lcn2), a natural cytoprotective factor, generated within the cell upon exposure to stressful conditions.	Bone marrow-derived stem cells (BMSCs) from rat bone marrow (4–6 weeks old).	(i) Lcn2-expressing BMSCs showed a more potent defense against H_2_O_2_, hypoxia, and serum deprivation stresses, compared to control MSCs (ii) Moreover, Lcn2 expression in MSCs increased cell proliferation and adhesion to culture media by 45% on H_2_O_2_ exposure, compared to control MSCs. (iii) Lcn2-expressing BMSCs showed normal multipotency into different cell lineage with mild potentiation of adipogenic lineage compared to the control group.	(i) The antioxidant effect of Lcn2 expression is due to ROS scavenging activity, associated with upregulation of antioxidant enzymes' genes, such as SOD. (ii) This antioxidant effect is associated with an antiapoptotic property as indicated by the diminished number of apoptotic cells on oxidative stress exposure, compared to the control group.
Fan et al. [38]	Alpha-phenyl-t-butyl nitrone (PBN) at 800 *μ*M and NAC at 5 mM.	Mesenchymal stem cells (MSCs) from mice embryos.	(i) Cultured MSCs with extracellular matrix from mouse embryonic fibroblast (MEF-ECM) under hypoxic conditions (2% O_2_) showed greater proliferation, lower generation of ROS, and increased chromosomal stability compared to the control group, cultured on plastic plates under normoxic conditions. (ii) To further diminish chromosomal instability and based on the increased DNA damage on H_2_O_2_ exposure, antioxidant supplementation to the culture media significantly reduced the number of DNA micronuclei and karyotyping abnormalities. The use of both (antioxidants and MEF-ECM) in the initial isolation of cells from the marrow increased purity and normal cell karyotyping.	Although authors did not investigate the underlying mechanisms for antioxidants' effects, they suggested that their findings can be attributed to the ability of both PBN and NAC to trap free radicals. Moreover, NAC serves as a precursor for glutathione, an intracellular antioxidant molecule.
Wang et al. [78]	2-Vinyl-8-hydroxyquinoline derivatives.	Mesenchymal stem cells (MSCs) from rat bone marrow.	In general, 2-vinyl-8-hydroxyquinoline derivatives had a positive effect on MSCs proliferation in a dose-dependent manner.	2-Vinyl-8-hydroxyquinoline derivatives are phenol compounds that perform their antioxidant activity through reaction of their hydroxyl group with free radicals.
Choi et al. [39] and Mekala et al. [79]	Ascorbic acid-2-phosphate (AAP) at 0, 5, 50 250, 500 mM.	Mesenchymal stem cells (MSCs) from adult human bone marrow [39] and human umbilical cord blood-derived stem cells (hUCB-SCs) from umbilical vein [79].	(i) Ascorbic acid significantly increased the proliferation of MSCs/hUCB-SCs, compared to the control group (with the highest proliferation rate at 250 mM). It had no effect on cellular antigenic expression and differentiation. (ii) Moreover, AAP significantly increased osteogenic differentiation at 50 mM (highest calcium deposition at this concentration). In contrast, a significant induction of adipogenic differentiation with oil droplets formation was noted at 250 mM and higher.	(i) AAP improved the amount of collagen production per cell and increased the amount of calcium (at 50 mM) and oil deposition (at ≥250 mM), enhancing MSCs/hUCB-SCs differentiation. (ii) The positive effect of AAP on cellular proliferation is dose-dependent (highest at 250 mM and decreases with higher doses due to inhibitory effect on glycosaminoglycan formation).
Ko et al. [80]	PEG-catalase (200 *μ*g/mL) and NAC (1 mM).	Human umbilical cord blood-derived stem cells (hUCB-SCs) from umbilical vein.	(i) Exposure to genotoxic stress (H_2_O_2_) in culture media caused a more significant reduction in cellular proliferation and DNA synthesis in hUCB-SCs, compared to control cells (cancer cells and human primary fibroblasts). (ii) Moreover, hUCB-SCs showed low resistance to oxidative stress with cellular senescence and apoptosis at H_2_O_2_ levels much lower than those of control groups.	Measuring the cellular antioxidant capacity showed that hUCB-SCs had a lower antioxidant capacity than control cells. To confirm that, antioxidant supplementation increased this capacity and diminished cellular damage upon exposure to oxidative stress.
Zeng et al. [81]	Edaravone (10 *μ*M), a clinically approved drug.	Human umbilical cord blood-derived stem cells (hUCB-SCs) from umbilical vein.	(i) Unlike the pro-oxidant (diethyl maleate), edaravone significantly reduced lipopolysaccharide (LPS)/H_2_O_2_-induced damage and increased stem cell viability (*p* < 0.05). (ii) In diabetic mice with severe combined immunodeficiency, only three mice died in the group, injected with (Gal/LPS and hUCB-SCs), compared to 50% loss in the group, injected with Gal/LPS only. (iii) Pretreatment with edaravone rescued all mice with potentiation of the hepatic cell regenerative power. Furthermore, it diminished the levels of cellular injury and proinflammatory markers in treated mice, compared to the control group.	(i) LPS/H_2_O_2_ challenge induced apoptosis by augmenting oxidative stress and increasing Bax/Bcl2 ratio. However, pretreatment with edaravone abolished these changes. (ii) Moreover, edaravone increased the expression of endogenous antioxidant enzymes (superoxide dismutase, catalase).
Rodriguez-Porcel et al. [41]	Tempol (SOD mimetic) at 0 to 10 mm/L concentration.	Rat cardiomyoblasts, transfected by a bioluminescence reporter gene for in vivo detection and transplanted into the myocardium, guided by high-resolution ultrasound.	(i) Cells, exposed to hypoxic/oxidative stress conditions during in vitro culturing, showed decreased cell viability with increased ROS production and NADPH-oxidase-1 expression, compared to the control group. These effects were significantly reduced after adding antioxidants in a dose-dependent manner. (ii) After transplantation into rat myocardium, antioxidant-treated cells showed significantly higher cell viability within the first three days of transplantation, compared to untreated cells.	Hypoxia induces oxidative stress by increasing the expression of NAD(P)H oxidase enzyme. Interestingly, adding antioxidant did not reduce NAD(P)H expression, suggesting that tempol reduces oxidative stress by neutralizing free radicals rather than decreasing their production.
Li et al. [40]	Homemade antioxidant cocktail consisting of 100 _M_L-ascorbate, L-glutathione, and *α*-tocopherol acetate.	Cardiac stem cells (CSCs) from the endomyocardial tissue of a patient undergoing a cardiac procedure.	(i) Cells, cultured under hypoxic conditions (5% O_2_) had a lower number of chromosomal abnormalities, compared to cells, cultured under normoxic conditions (20% O_2_). (ii) Unexpectedly, increased cytogenic abnormalities in number and severity were recorded in cells, cultured with antioxidant supplementation. (iii) Antioxidant supplementation, if excessive, may hinder the physiological roles of ROS in stem cells' proliferation and differentiation, which raises a new concept [reductive stress].	(i) Measuring c-H2AX foci (a marker of DNA breaks) showed a biphasic relationship between ROS levels and frequency of DNA breaks, that is, increased DNA damage occurs at low antioxidant/high ROS levels, while excessive suppression of ROS levels increases DNA damage. (ii) There is an optimal level of ROS in stem cells, above which genomic instability occurs due to ROS-induced DNA damage and below which DNA repair enzymes are not activated to maintain the DNA stability.
Takahashi et al. [82]	Ascorbic acid (10^4^ M/L) incubation for 12 days.	Human embryonic stem cells (ESCs).	(i) Ascorbic acid significantly increased ESCs differentiation into cardiac myocytes in a dose-dependent manner, as evidenced by increased expression of the cardiac specific gene (myosin heavy chain (MHC)). (ii) Ascorbic acid, independent of its antioxidative property, induced ESCs differentiation into cardiac myocytes. This was evidenced by the absence of this effect with other antioxidants, such as NAC, Tiron, and vitamin E.	Ascorbic acid increased the expression of cardiac muscle genes, such as GATA4, Nkx2.5, *α*-MHC, *β*-MHC, and atrial natriuretic factor (ANF), with subsequent cardiac-specific protein production.
Song et al. [43]	Selenium (20 or 50 ng/mL) and NAC (100 *μ*M).	Human embryonic stem cell- (ESC-) derived vascular progenitors.	(i) Following vascular differentiation of ESCs into vascular progenitor cells (CD34+ cells), a quiescent state of cellular proliferation developed with 41% of the cells in the G0 phase, upregulation of the G1 checkpoint inhibitor (p21) protein and downregulation of mitosis-related genes. (ii) Selenium increased cellular proliferation, reduced p21 expression, and decreased the number of cells in G0 phase of cell cycle. Moreover, it promoted the vascular differentiation of ESCs with no similar effect on endodermal or ectodermal potentiality. (iii) Using NAC totally inhibited the vascular differentiation of ESCs.	(i) Physiologically, ROS are produced during vascular differentiation mainly by NADPH oxidase and is responsible for this quiescent state. (ii) Selenium through increasing glutathione and thioredoxin activity moderately diminished ROS levels. (iii) However, NAC, through complete scavenging of ROS, abolished their physiological role in vascular differentiation.
Park et al. [45]	GV1001 [derived from human telomerase reverse transcriptase from 0 to 100 *μ*M.	Neural stem cells (NSCs) from mice embryonic brain (cortical tissue).	GV1001 significantly reduced H_2_O_2_ effects on NSCs including diminished cellular proliferation, migration and increased apoptosis. Interestingly, GV1001 itself had no effect on normal untreated cells.	(i) GV1001 has an ROS-scavenging activity, preventing lipid peroxidation and DNA damage. (ii) At the molecular level, GV1001 induced the expression of survival-related proteins and diminished the expression of apoptosis-related proteins.
Hachem et al. [46]	Cyclosporine A (CsA), brain-derived neurotrophic factor (BDNF), and thyrotropin-releasing hormone (TRH).	Neural stem cells (NSCs) from the spinal cord of transgenic adult female rats (spinal cord injury model).	(i) Pretreatment with BDNF for 48 hours (before H_2_O_2_ exposure) significantly increased NSCs viability and decreased intracellular ROS accumulation, compared to the control group. However, CsA and TRH-treated cells showed no significant changes from the control group. (ii) Interestingly, BDNF-treated cells showed no changes in cellular proliferation and differentiation compared to the control group.	The neuroprotective effect of BDNF is exerted through its ROS-scavenging activity and induction of antioxidant enzymes, such as GR and SOD. Moreover, significant reductions in apoptotic features were noted in BDNF-treated cells, compared to the control group.
Song et al. [52]	Melatonin (100 nM).	Neural stem cells (NSCs) from mice embryonic cortical tissue.	(i) Melatonin significantly reduced LPS-induced toxicity and apoptosis of NSCs through reducing nitric oxide (NO) production and inducing antioxidant enzymes. (ii) Furthermore, it maintained the neurosphere size in NSCs, treated with LPS. (iii) Melatonin increased cell survival by activating PI3K/Akt pathway. This was confirmed by the addition of wortmannin (a PI3K inhibitor), which inhibited Nrf2 expression and subsequent antioxidant activities.	(i) Melatonin increased the expression of multiple transcriptional factors, involved in NSCs proliferation, self-renewal, and differentiation, such as orphan nuclear receptor TLX and fibroblast growth factor receptor-2. (ii) Melatonin upregulated the expression of nuclear factor-erythroid 2-related factor 2 (Nrf2), responsible for downstream activation of antioxidant enzymes' genes.
Sun et al. [59]	N-Acetyl-L cysteine (NAC) at 2 mM and ascorbic acid-2-phosphate (AAP) at 0.2 mM in comparison to the effect of hypoxia.	Adipose-derived stem cells (ADSCs) from human adipose tissue.	ADSCs, grown in media, supplemented by antioxidants or under hypoxic conditions (5% po2), showed a more significant increase in cell proliferation and a decrease in doubling time than the control group, supplemented by fibroblast growth factor-2. Moreover, cytometric analysis showed that cells, cultured in antioxidant-supplemented and hypoxic media, had a greater proportion of cells in S1 phase of the cell cycle with diminished G0/G1 phase cells, compared to the control group.	In antioxidant-supplemented media, PCR showed diminished levels of cyclin-dependent kinase inhibitors (CDK: important cell cycle regulators that control entering S1 phase), with enhanced expression of stemness-related genes, compared to the control group.
Lyublinskaya et al. [11]	Tempol (1-2 mM), NAC (5–20 mM), and resveratrol (20–40 *μ*M).	Endometrial stem cells, isolated from desquamated endometrium of menstrual blood and ADSCs from adipose tissue.	(i) Reactive oxygen species are important regulators of stem cell self-renewal and proliferation upon exit of the quiescent stage. (ii) Using a synchronized cell in G0 phase, there was a transient increase in ROS levels upon stimulation of cell proliferation and during initial stages of DNA synthesis. (iii) Adding antioxidants to the medium after proliferation induction and before initiation of S1 phase blocked S1 transition. Antioxidant did not have the same effect when added after S1 initiation.	(i) Cells, treated with antioxidants, showed expression of the proliferative marker (Ki-67), which is absent in the nucleus of quiescent cells, indicating that the cell left the quiescent state and was arrested in G1 phase. (ii) Antioxidants, through dose-dependent reduction of ROS levels, can be used to control cellular proliferation during in vitro culturing.
Yang et al. [65]	Fullerol (a polyhydroxylated fullerene) at 0.1, 0.3, 1, 3, and 10 *μ*M.	Human adipose-derived stem cells (ADSCs).	(i) Fullerol enhanced the osteogenic differentiation of ADSCs, as indicated by increased expression of osteogenic markers (Runx2, OCN, and alkaline phosphatase) and mineralization. (ii) Moreover, fullerol (at all concentrations) reduced ROS levels in the osteogenic culture media.	Fullerol exerted an antioxidant effect on ADSCs through potentiating the expression of the transcription factor FoxO1 and its downstream genes (Runx2 and SOD2), which promote ROS scavenging and osteoblastic differentiation.
Yu et al. [83]	L-Ascorbic acid 2-phosphate (AAP) at 250 *μ*M.	Adipose-derived stem cells (ADSCs) from the subcutaneous adipose tissue from a female patient, undergoing abdominoplasty.	(i) Ascorbic acid significantly increased ADSCs proliferation, preserved cellular stemness and increased the potentiality for adipogenic, hepatic, neural and osteogenic differentiation. (ii) In AAP-induced cell sheet, there was a significant increase in the genetic expression and secretion of collagen, laminin, and fibronectin proteins. (iii) The AAP-induced cell sheet improved wound healing in a murine wound model, which indicates the possibility of ADSCs differentiation in a non-mesenchymal lineage.	(i) Adding AAP to ADSCs increased the expression of stemness-related proteins. (ii) Using other antioxidants, such as NAC did not show an increase of stemness markers. In contrast, adding a collagen synthesis inhibitor abolished AAP-induced overexpression of stemness proteins, indicating that the involved mechanism in AAP action is collagen synthesis, not ROS scavenging.
Wang et al. [66]	NAC and AAP at 3 mM and 0.2 mM, respectively (for 20 hours).	Human ADSCs from 10 different human patients.	(i) Pretreated MSCs with antioxidants showed less apoptosis and lower caspase-3 levels upon exposure to advanced glycosylation end-products (AGE), compared to the control group. (ii) The effects of NAC and AAP were significantly amplified after the addition of miRNA-223 mimetics and were significantly abolished by miRNA-223 inhibitors.	Antioxidants reduced ROS generation and apoptosis, induced by AGE. This can be explained by the effect of both on miR-223 (a regulator of intracellular apoptotic singling through modulation of fibroblast-like growth factor receptor-2 protein levels.
Drowley et al. [72]	N-Acetyl-L cysteine at 10 mM in comparison to the pro-oxidant (diethyl maleate) at 50 *μ*M.	Muscle-derived stem cells (MDSCs) from the skeletal muscle of 3-week-old female mice.	(i) In comparison to control cells, NAC-treated cells showed increased survival and differentiation into myotubes upon exposure to oxidative (H_2_O_2_) or inflammatory stress (tumor necrosis factor). (ii) In failed mice hearts, injection with NAC-treated cells significantly improved systolic and diastolic function on echocardiographic assessment, compared to injection with untreated and DEM-treated cells. Moreover, scar tissue formation was significantly lower in NAC-treated cells than untreated cells.	(i) Increased cell survival after NAC treatment is probably related to stimulation of mitogen-activated protein kinases (MAPK) and extracellular signal-regulated kinase (ERK), kinase families involved in cellular survival and proliferation. (ii) Interestingly, a similar increase in CD31+ endothelial cells in NAC-treated and untreated MDSCs was observed.
Aliakbari et al. [74]	Catalase (40 mL) and *α*-tocopherol (200 mL).	Spermatogonial stem cells (SSCs) from neonatal male mice testis.	Antioxidant supplementation of cryopreserved SSCs reduced oxidative damage to membranes and organelles and increased cell survival in a dose-dependent manner.	Catalase and *α*-tocopherol reduced ROS generation in treated cells, compared to control cells. Moreover, antioxidant-treated cells showed an increased expression of the anti-apoptotic BcL-2 gene with decreased expression of the pro-apoptotic BAX gene, compared to the control group.

ADSCs: adipose-derived stem cells; CAT: catalase; DEM: diethylmaleate; GSH: glutathione; HSCs: hematopoietic stem cells; iPSCs: induced pluripotent stem cells; MDSCs: muscle-derived stem cells; NAC: N-acetyl cysteine; NSCs: neural stem cells; SCC: spermatogonial stem cells; SOD: superoxide dismutase; ROS: reactive oxygen species.

## Abbreviations

ADSCs: Adipose-derived stem cells
BMSCs: Bone marrow-derived mesenchymal stem cells
CAT: Catalase
DEM: Diethylmaleate
DFO: Deferoxamine
GSH: Glutathione
hPDLCs: Human periodontal ligament cells
HSCs: Hematopoietic stem cells
iPSCs: Induced pluripotent stem cells
MDSCs: Muscle-derived stem cells
NAC: N-acetyl cysteine
NSCs: Neural stem cells
PBP: Phosphate buffered saline
SCCs: Spermatogonial stem cells
SOD: Superoxide dismutase
ROS: Reactive oxygen species.
